# Supplementary material for: Spike-timing dependent plasticity partially compensates for neural delays in a multi-layered network of motion-sensitive neurons
Source: PLoS Comput Biol. 2023 Sep 6;19(9):e1011457. doi: 10.1371/journal.pcbi.1011457 (PMC10506708; doi:10.1371/journal.pcbi.1011457)
Supplement: Attachment — Submitted filename: Response to reviewers.docx [file pcbi.1011457.s001.docx]

**Sexton, Burkitt & Hogendoorn: Response to Reviewers**

We thank both reviewers for their valuable feedback, and appreciate the time taken to provide helpful comments on the improvement of the manuscript. We have made revisions to address the concerns raised, and believe the manuscript has been greatly improved as a result. We address the specific comments raised by reviewers below.

**Reviewer 1**

***1.1)*** *Overall I found the paper very interesting and quite well conceived. My remarks are mostly about minor details. This work uses a simple neuronal model (no homeostasis, inhibition...) but demonstrates the ability of STDP learning for compensating for delays during motion perception. Their related work mentions no similar work in that domain. The result part is thorough, but could benefit from some more quantitative analysis as it can be difficult to gather all the information from the plots only.*

We thank the reviewer for this suggestion and have revised the results section to aid visualisation of the main findings. Specifically, we have revised Fig 5 by adding a second subplot that groups the receptive-field shift magnitudes by membrane time constant, in addition to being grouped by layer. This makes it easier to directly quantitatively evaluate the effect of membrane time constant on receptive-field shift at high velocities.

Additionally, we have revised Fig 8, which now includes a subplot that demonstrates the effect of STDP-training on realtime stimulus localisation across all velocities (replacing Fig 9B in the original manuscript), as well as a subplot that quantifies temporal-alignment of layers across all velocities, to accompany the qualitative description of this result.

***1.3)*** *All the results are presented on simulated inputs. It would be a great addition to try the model on real inputs or a standard motion dataset.*

We agree with this suggestion and have added a comment regarding this to the discussion section:

**LINE 512:**

“In this work, we have included a simplified model of velocity-selectivity in which neurons are active in response to a singular, one-dimensional input velocity. A more biologically realistic extension of this network would be to determine neural activity based on a characteristic tuning curve over a range of velocities. In addition, the one-dimensional nature of the network prevents its application to standard motion datasets, which are typically two-dimensional and contain motion energy at various velocities. Future work could therefore extend the network to two-dimensions, and implement a global read-out mechanism that forms representations of a dynamic visual scene based on the weighted, simultaneous activity of all velocity-tuned networks. ”

***1.4)*** *My major concerns are on the related work part. It presents very few papers on the subject except biological papers that focus on the biological effect of motion-lag compensation.
There is some literature missing on the subject of learning motion representations with spiking neural networks, such as:
- Unsupervised Learning of a Hierarchical Spiking Neural Network for Optical Flow Estimation: From Events to Global Motion Perception
Federico Paredes-Vallés, Kirk Y. W. Scheper, Guido C. H. E. de Croon
- Learning heterogeneous delays in a layer of spiking neurons for fast motion detection
Antoine Grimaldi and Laurent U Perrinet
- Spike timing-based unsupervised learn-ing of orientation, disparity, and motion representations in a spiking neural network.
T. Barbier, C. Teulière, and J. Triesch
Even though those papers do not focus specifically on the work of motion lag compensation, they present important mechanisms for motion tuning. If this is the only model that specifically implements a motion lag compensation framework, I would mention this explicitly.*

We thank the reviewer for their specific suggestions on related work and agree that this field of work is useful to discuss in this manuscript. We have revised the introduction to include studies that utilise STDP as a mechanism underlying motion-sensitivity:

**LINE 118:**

**“**Other modelling work has implemented STDP as a learning mechanism in multilayer spiking neural networks (SNNs), with particular focus on its capacity to generate motion direction [18] and velocity [19] selectivity in simulated neurons. Although Burkitt et al. [15] did not address how velocity-sensitivity was formed among the neurons in their simulation, they showed how STDP can alter the structure of feedforward connectivity *between* those neurons, in such a way that downstream neurons begin to respond earlier to motion stimuli. To our knowledge, no work to date has addressed the combined effect of STDP-driven receptive field shifts [11,15] across multiple levels of a hierarchical network.”

We also now discuss papers that have addressed motion encoding in spiking neural networks as well as networks that address motion selectivity via delay selection (also see response to comment 1.5).

**LINE 505:**

**“**Previous work incorporating delay selection has addressed its capacity to generate motion selectivity in SNNs [27,28]. Related work has also highlighted the role of STDP as a mechanism by which motion-selectivity may arise [18,19,29]. In this work, we have not modelled the formation of motion encoding, but for simplicity have taken as a starting point discrete populations of neurons endowed with velocity-selectivity. An interesting avenue for future exploration would therefore be to also incorporate the formation of velocity-selectivity into the learning process, potentially via STDP-driven selection of specific feedforward delays.”

***1.5)*** *line 139: Delays in biological model are not necessarily constant for each neuron. What problems, if any, do you expect for a model with variable delays? What could be done to address such problems?*

We agree with the reviewer that the question of variable delays/delay selection is relevant to this discussion, and have incorporated additional literature demonstrating that STDP can itself help with selecting from inputs with different delays. We now discuss address this in the Discussion:

**LINE 494**

“Additionally, incorporating multiple delay lines between neurons could potentially increase the capacity of RTTA to emerge in the network. Previous research has demonstrated how STDP may underlie ‘delay selection’: the selective potentiation of specific connections between neurons from a range of connections with varying delays [23,24], thought to occur early during development [25] and underlie processes requiring a high degree of temporal coherence between neurons [23]. Although we have modelled a single, fixed transmission delay between neurons, it would be possible to extend the model to incorporate a subset of connections between each pair of neurons, across which transmission delay time is varied. This would increase the range of adaptation in the temporal dynamics of the network [26], potentially expanding the range of velocities across which RTTA is observed. However, it is possible that such a network would still require some form of feedback in order to select delays that yield the highest degree of coherence across regions.”

***1.6)*** *Line 210: The training occurs for around 20 seconds. This seems like a very short time compared to real biological training time. Please comment.*

We agree that the learning process would take place over a longer period biologically, however the training duration is not intended to faithfully capture the biological evolution of the receptive field shifts we describe. We chose a training duration that would both limit computation time and produce asymptotic receptive field shifts, given the learning rate used. It would definitely be possible to lower the learning rate and extend the training duration. However, as the findings are based on asymptotic receptive field shift magnitudes, this should have no bearing on the results obtained. Essentially it merely allows us to “fast-forward” to the equilibrium network state that would eventually be achieved. We have added a comment on this in the Method:

**LINE 212:**

“The training procedure takes place over $N_{t}$ *=* 20,000 timesteps (equivalent to 20 simulated seconds). Although this duration is shorter than a true biological training window is likely to be, our analysis focuses on the asymptotic properties of the network, and the results should therefore be independent of how long the network takes to reach that asymptote. Choosing a relatively high learning rate and short training time allowed us to save computation time.”

***1.7)*** *Fig 5: It is difficult to see the difference in shift between the 4 plots. Please provide a quantitative analysis.*

As per our response to comment 1.2, we have included a new subplot in Fig 5 that groups receptive-field shifts by membrane time constants to better show quantitative differences across these values.

***1.8)*** *You address the motion tuning as a dual layer phenomenon (lower cells encode position, higher cells encode motion). Have you thought about lower cells with synapses of various delays in order to encode motion instead? This could be worth discussing.*

We agree with the reviewer that this point is worth discussing, and have added a paragraph on this to the discussion:

**LINE 505 (*repeated from comment 1.4)*:**

**“**Previous work incorporating delay selection has addressed its capacity to generate motion selectivity in SNNs [27,28]. Related work has also highlighted the role of STDP as a mechanism by which motion-selectivity may arise [18,19,29]. In this work, we have not modelled the formation of motion encoding, but for simplicity have taken as a starting point discrete populations of neurons endowed with velocity-selectivity. An interesting avenue for future exploration would therefore be to also incorporate the formation of velocity-selectivity into the learning process, potentially via STDP-driven selection of specific feedforward delays.”

**Reviewer 2**

***2.1)*** *The plasticity mechanism proposed only relies on a simple weight normalization, and remains blind to the true speed of the stimulus (overestimation for low velocities, underestimation for high velocities, with an "optimum" at around 0.8 cycle/s in fig. 9). As explained in the discussion, this mechanism alone is not enough to implement a true temporal alignment.*

We agree with the reviewer that the effect of ‘perfect compensation’ is dependent on both the velocity and temporal delay between layers, and have edited the original manuscript to acknowledge this more clearly. We address this point specifically in the Discussion:

**LINE 468:**

“We observed that the magnitude of the forward shift in the encoded position of the motion stimulus was in some cases sufficient to lead to realtime representations of location that were ahead of or in line with the stimulus (Fig 8A), despite accumulated transmission delays in the network being as high as 100ms for layer six. It must be noted that this effect is dependent on the specific transmission delay and input velocity (as well as the membrane time constant and stimulus intensity). This is because the STDP mechanism modelled is insensitive to the specific transmission delay or input velocity. The observed increase in temporal alignment of positional representations across layers (Figure 8B) has the same dependency on delay time and velocity. In order to achieve temporal alignment across a broader range of velocities and transmission delays, it may be necessary to incorporate a feedback mechanism into the model, to allow extrapolated representations at higher levels to be adjusted based on their correspondence with lower-level activity.”

***2.2)*** *More problematically, the way the authors pretend their model may reflect the flash-lag effect (FLE) illusion is an overstatement, and the figure 2 is clearly misleading to that prospect. One can not pretend to propose a mechanistic model of the flash lag effect by using different weights for different stimuli (!).*

We thank the reviewer for highlighting a potential confusion underlying how we have modelled several subpopulations of neurons, each with an associated set of weights. We have made revisions throughout the method section to make this clarification more explicit:

**LINE 148:**

“The network thus described is replicated across *N*_v_ velocity-tuned subpopulations, with activity in each subpopulation determined in an all-or-none fashion depending on the input stimulus; only the subpopulation tuned to a particular stimulus velocity will be activated, all others remain inactive. We also include a zero-velocity/static subpopulation of neurons that are activated by stationary input. This ‘static network’ can be regarded as a subpopulation of neurons tuned to stimulus position but with no velocity tuning. In this arrangement, the network as a whole is comprised of *N*_v+1_ subnetworks with their own separate connection weights.”

**LINE 216:**

“Following initialization of the network weights, a moving input stimulus traverses the input for the full 20 seconds, evoking activity in the subpopulation tuned to the stimulus velocity. Weights within that population evolve according to STDP at each timestep – no STDP is applied to the inactive subpopulations.”

**LINE 235:**

“After the process of STDP learning, spiking responses are compared between the (trained) velocity-tuned networks and the (untrained) static network. Specifically, responses of each velocity-tuned network to motion input at their preferred velocity are compared with responses of the static network to stationary flashes.”

In addition, we have also removed reference to ‘FLE magnitudes’ in the results section, and now emphasise more clearly within the Discussion that we are not proposing a mechanistic explanation of the flash-lag effect:

**LINE 536:**

**“**In order to quantify the effect of STDP on instantaneous position representations of flashed and moving stimuli, we utilised an approach similar to a typical flash-lag effect (FLE) paradigm. In the FLE illusion, observers report a briefly flashed stimulus to lag behind a moving stimulus, despite their physical alignment [5]. A number of hypotheses regarding the underlying causes of the FLE have been proposed (for reviews see [33,34]), and our aim is not to advocate for any particular explanation of the FLE. The network described here, comprising only of feedforward connections and a simple learning mechanism, is not intended to capture the rich variety of mechanisms underlying perceptual illusions such as the FLE.”

***2.3)*** *In conclusion, the authors have provided a timely analysis of an important receptive field shift effect that is shown to cumulate over layers. However, the authors tend to oversell their results and falsely pretend that it compensates for the time delays (this is only true in a tiny interval), and may explain more high-level features like the flash lag effect.
For that reason, the paper can not be accepted in the present writing. Both the introducion and the descussion need to be rewritten to reflect the limited scope of the results.*

We thank the reviewer for their constructive feedback and have revised the manuscript in line with their suggestion, to provide more precision in our description of the capacities of the network. Across the revised manuscript, the effect of STDP-training is now presented as reducing the overall lag between the stimulus and its represented position, with limited reference to specific delay compensation. As per our response to comment 2.1, we have made the velocity-dependence of the effect more explicit in order to better highlight this limitation of the network in its current form. As per our response to comment 2.2, we have also made several changes to make explicit the fact that we are not proposing a mechanistic explanation for the flash-lag effect.

***2.4)*** *Figure 2 is clearly misleading and should be removed.*

We agree that the original manuscript could be improved with a more explicit description of how the different subpopulations of neurons are modelled, and have made revisions to this effect as per our response to comment 2.2. In addition, our changes to Fig 2 are also intended to make this point more clear. We have edited the figure so that the static network and velocity-tuned network responses are separated across subplots. We also include a more explicit description of the simulation procedure in the figure caption:

**LINE 252:**

“**Fig 2. A.** Example input layer activity in the static network, in response to a stationary flash. *TOP:* firing rate response of input layer neurons. *BOTTOM*: Spikes generated based on these underlying firing rates, which are fed forward to layers 2 and above. **B.** Example of input layer activity in a velocity-tuned subpopulation, in response to a motion stimulus. Top and bottom panels indicate firing rates and spikes, respectively, as in (A). **C.** Spiking distribution peak differences between static network responses to flash and velocity-tuned network responses to motion. Top: Spiking locations across neurons in a single layer (vertical lines) and timepoint are convolved with a Gaussian kernel to produce a continuous probability density function (PDF), in both the static network (blue) and the velocity-tuned network (red). A measure of spiking peak difference is calculated based on the angular error between peaks (dotted lines). Bottom: Spiking peak differences are recorded across 200 trials, and a von Mises distribution (red curve) is fitted to compute the average spiking peak difference.”

With these revisions, we believe it is important to retain Fig 2 in the revised manuscript, as it serves to illustrate the methodology used in measuring spiking responses across the static and velocity-tuned subnetworks.

***2.5)*** *Also fig. 8 should be removed for it suggest to a distracted reader that the temporal alignment is effective at all speed.*

We have removed the original Fig 8, and have now included similar plots of the single unit response across time, for all velocities, as a subplot (Fig 8C).

***2.6)*** *Moreover, a new figure should clearly quantify, on a layer by layer basis, the shift in temporal alignment in function of the stimulus speed (for fig.9 is quite difficult to read from that perspective)*

We thank the reviewer for this suggestion. The revised Fig 8 has replaced the original Fig 8 and Fig 9. We have removed the plots that showed the peak of the population response across time, relative to stimulus location, and instead plot this across all velocities, for a single timepoint, in Fig 8A. This new figure more clearly captures the relative differences between layer-based representations and the stimulus location. In addition, we include an additional plot (Fig 8B) which quantifies the degree of alignment across layers, for all velocities.
