# Supplementary material for: Spike-timing dependent plasticity partially compensates for neural delays in a multi-layered network of motion-sensitive neurons
Source: PLoS Comput Biol. 2023 Sep 6;19(9):e1011457. doi: 10.1371/journal.pcbi.1011457 (PMC10506708; doi:10.1371/journal.pcbi.1011457)
Supplement: Attachment — Submitted filename: Response to reviewers2.docx [file pcbi.1011457.s002.docx]

**Sexton, Burkitt & Hogendoorn: Response to Reviewers**

**Reviewer 2**

***2.1)*** *I appreciated the effort made by the authors in significantly reworking the introduction and conclusions of this paper, which has gained clarity compared to the previous version. While clarifying their model and results, it seems to me that in their conclusions, the authors do not sufficiently discuss certain important limitations.*

We thank the reviewer for the time taken to assess the revised manuscript, and for their valuable comments and feedback. We have addressed these concerns in revisions to the manuscript, which are detailed below.

***2.2)*** *Firstly, the proposed model uses a large number of synaptic relays and layers of neurons for the processing of visual data. As stated, each speed selectively activates a sub-network and inhibits the rest. How can such a system claim to process a large number of directions and speeds (beyond those analyzed in the paper)? It appears highly improbable that the visual system dedicates a specialized system to each direction/speed, considering that the number of considered layers (six synaptic contacts) can account for the entirety of rapid visual processing (Thorpe et al., 1996). Would it be possible to do away with these specialized sub-networks to achieve more efficient and economical visual processing?*

We agree with the reviewer that the motion processing modelled is simplified and therefore not optimised to process natural inputs, and have added text to the Discussion to highlight these simplifications and how they could be addressed in future research:

**LINE 524:**

*“*In this work, we have included a simplified model of velocity-selectivity in which neurons are active in response to a singular, one-dimensional input velocity. This approach was adopted in line with the specific goal of addressing the effect of stimulus velocity on STDP-driven receptive field shifts, as a proof-of-principle, rather than deriving a model that captures the full extent of visual motion processing. Indeed, the model described here is likely to be largely inefficient for processing the full range of directions and speeds to which the visual system is sensitive. In addition, the one-dimensional nature of the network prevents its application to standard motion datasets, which are typically two-dimensional and also contain motion energy at various velocities. A more biologically realistic extension of this network would be to determine neural activity based on a characteristic tuning curve over a range of velocities. Future work could also extend the network to two-dimensions, and implement a global read-out mechanism that forms representations of a dynamic visual scene based on the weighted, simultaneous activity of all velocity-tuned networks.”

***2.3)*** *Secondly, the case of static inputs is superficially addressed. With the proposed model, temporal alignment is fine for targets with a speed of 0.8 cycles/s, but in principle, it should also be fine for stationary targets. Can the authors confirm whether a static target would be correctly represented in all layers, just like a moving target? Does the widening of the receptive field with layers for low speeds pose a problem? If so, how can it be overcome? How can a system be proposed that accurately processes both static and moving targets across layers? Please elaborate on your conclusions and discussion regarding these two points.*

We thank the reviewer for their suggestion regarding the clarification that a stationary input will be correctly represented across all layers in our network, and have added a comment on this to the Discussion:

**LINE 491:**	“Note that for static inputs, such as a stationary object, extrapolation mechanisms would be not be required for RTTA to occur, given that the location of the represented stimulus is unchanging across time [22]. In the case of the static network presented here, representations at each layer would align with the position of a sustained, stationary stimulus (assuming enough time for each layer to receive input layer activity), given that no STDP training was performed on the static network weights (replicating the case in which STDP effects are spatially symmetric).”

Regarding the question of whether widening receptive fields pose a problem for representations at each layer, because we treat the peak of the population distribution as the represented stimulus location, widening activity distributions at successive layers will not affect the represented location that is computed. Regarding the question of how the model can accurately represent both static and moving targets: there is no conceptual difference between the models tuned to moving targets (nonzero velocities) and static targets (zero velocity). All models start out tuned to the original physical position. However, for the moving stimuli, STDP causes asymmetric potentiation around this position which causes receptive field shifts. For the static model, no asymmetric stimulation would take place, and no systematic shifts would therefore be expected.

Because no asymmetric learning was expected in the static case, we did not expose the static model to learning, but rather used the initial state of the untrained network as a proxy.

We now clarify this point further in the Method and Discussion:

**LINE 228:** “As mentioned, neurons in the static network are regarded as being responsive to localised, stationary inputs, rather than being activated by a specific velocity. Although these neurons would be subject to STDP in a biological setting, a balanced STDP function and non-directional inputs would result in spatially symmetric connection weights, essentially replicating the initial state of the network. We therefore do not perform any training procedure for this network – the weights for this subpopulation are initialised as described above and do not undergo any STDP learning.”

**LINE 493:** “In the case of the static network presented here, representations at each layer would align with the position of a sustained, stationary stimulus (assuming enough time for each layer to receive input layer activity), given that no STDP training was performed on the static network weights (replicating the case in which STDP effects are spatially symmetric).”

***2.4)*** *Minor point: Figure 2 remains unclear and should be improved, perhaps by reversing the black/white contrast. In the figure caption, it is difficult to understand how many layers were used to measure the temporal shift (layer 2 and above??). Some axes lack units.*

We thank the reviewer for their suggestions to improve Figure 2 and for pointing out a potential confusion in the interpretation of the figure caption. We have reversed the black/white contrast in the first two panels. The caption for Fig 2A has been updated as per below:

**LINE 257:**	“**Fig 2. A.** Example input layer activity in the static network, in response to a stationary flash. *Top:* Firing rate response of input layer neurons. *Bottom*: Spikes generated based on these underlying firing rates, which are transmitted via feedforward connections to layer 2, and so on.”

The figure is intended to illustrate some example (and arbitrary) activity in the static network and a velocity-tuned network, to accompany the text description of the input layer firing rate activity as a Gaussian centered on the stimulus position, from which spikes are generated according to a Poisson process, and to illustrate how this process unfolds across time. Panel C (*Top)* is intended to illustrate a series of spikes at a single timepoint, and how the peak of the spiking distribution is estimated from this. In the actual analyses, this approach was applied to every layer and timepoint.
